# [Retracted] miR-5590-3p inhibits the proliferation and metastasis of renal cancer cells by targeting ROCK2 to inhibit proliferation, migration and invasion

**DOI:** 10.3892/ol.2026.15749

**Published:** 2026-07-06

**Authors:** Queling Liu, Anyi Zhu, Weiyin Gao, Fu Gui, Yan Zou, Xiaocheng Zhou, Zhengdong Hong

Oncol Lett 24: 377, 2022; DOI: 10.3892/ol.2022.13497

Following the publication of this paper, it was drawn to the Editor's attention by a concerned reader that, regarding the colony formation assay data shown in Fig. 4C on p. 9, the images shown for the Untreated and ROCK2 experiments with the A496 cell line were strikingly similar, even though the intensity of the images differed. In addition, the ‘Tumor/1’ and ‘Tumor/2’ data panels in Fig. 1D were found to contain an overlapping section, and three different pairs of overlapping data panels were identified comparing the Transwell invasion assay data in Figs. 3D and 4E, suggesting that these data were derived from the same original sources, where different experiments were intended to have been portrayed.

Upon investigating the data in this paper in the Editorial Office, the concerns of the reader were shown to be well-founded. Therefore, owing to the number of overlapping data panels identified in Fig. 2, the Editor of *Oncology Letters* has decided that this paper should be retracted from the Journal on account of a lack of confidence in the presented data. The authors were asked for an explanation to account for these concerns, but the Editorial Office did not receive a reply. The Editor apologizes to the readership for any inconvenience caused.

